# Biomaterial-Based Approaches for Regeneration of Periodontal Ligament and Cementum Using 3D Platforms

**DOI:** 10.3390/ijms20184364

**Published:** 2019-09-05

**Authors:** Chan Ho Park

**Affiliations:** 1 Department of Dental Biomaterials, School of Dentistry, Kyungpook National University, Daegu 41940, Korea; 2 Institute for Biomaterials Research and Development, Kyungpook National University, Daegu 41940, Korea

**Keywords:** biomaterials, tissue engineering, regenerative medicine, periodontal ligament, cementum, periodontal tissues

## Abstract

Currently, various tissue engineering strategies have been developed for multiple tissue regeneration and integrative structure formations as well as single tissue formation in musculoskeletal complexes. In particular, the regeneration of periodontal tissues or tooth-supportive structures is still challenging to spatiotemporally compartmentalize PCL (poly-ε-caprolactone)-cementum constructs with micron-scaled interfaces, integrative tissue (or cementum) formations with optimal dimensions along the tooth-root surfaces, and specific orientations of engineered periodontal ligaments (PDLs). Here, we discuss current advanced approaches to spatiotemporally control PDL orientations with specific angulations and to regenerate cementum layers on the tooth-root surfaces with Sharpey’s fiber anchorages for state-of-the-art periodontal tissue engineering.

## 1. Introduction

### 1.1. The Characteristics of Periodontal Ligament and Cementum in the Periodontal Complex

**Figure 1:**
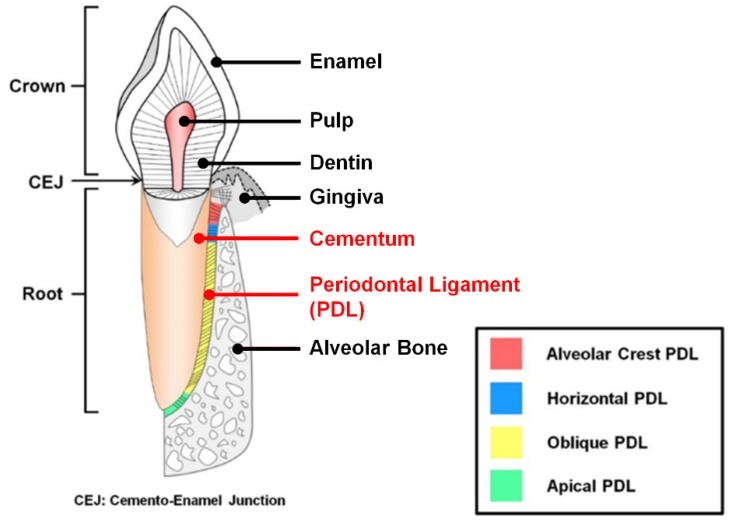
Illustration of tooth structure and tooth-supportive complex. Four different types of periodontal ligament (PDL) fibers are typically categorized between the cementum on the tooth-root surface and the alveolar bone. Adapted with permission from the reference [8].

The periodontal complex has hierarchically compartmentalized architectures with structural integrations of fibrous connective tissues and mineralized tissues surrounding tooth structures [1]. As tooth-supportive structures, three different tissues are typically composed like cementum (the mineralized layers on the tooth-root surface), periodontal ligaments (PDLs; the fibrous connective tissues between cementum and bone surface with specific orientations to the tooth-root surfaces), and alveolar bone (the mineral construct to sustain teeth and tooth-associated tissues with alveolar sockets) (Figure 1) [2,3,4]. Of the periodontium, a major component of the function of the alveolar bone is to appropriately position teeth and continuously remodel structures by generating mechanical responses against compressive or tensile forces that are exerted by various external stimulations [5,6,7]. These mechanical transmissions of masticatory forces around teeth could be spatially generated by integrating fibrous connective tissues or PDLs with appropriately physical tautness, so PDL anchorages between cementum and alveolar bone surfaces within compartmentalized tissue interfaces should be required for the functionalized periodontal complex [3,5,8]. To form the tissue integrations as tooth-supporting constructs, Sharpey’s fibers, which are the terminal ends of principal PDL fibers, crucially contribute to inserting and anchoring into the cementum and the mineralized layer of bone surfaces [1,9,10]. By integrating these multiple tissues, PDL fibrous bundles can mainly provide supportive, remodeling, sensory, and nutritive functions during mastication and occlusion [11]. In particular, oblique or perpendicular orientations of PDLs to the tooth-root surface when viewed coronally can play pivotal roles in generating various mechanical or biological responses during mastication and occlusion to protect teeth and bone structures [4,8,9].

Figure 1 shows that integrated fibrous connective tissues (PDLs) are categorized into four typical types: alveolar crest (radiated bundles), horizontal (perpendicular bundles), oblique (oblique bundles coronal-attached to bone), and apical (radiated bundles) fibers by generating optimal mechanical resistances or biological responses under various physiological loading environments [3,9]. In particular, the oblique PDL group, which spatially constitutes approximately 70% of the complex, mainly resists vertical and intrusive forces during mastication applications, while other typical PDL groups generate systemic responses to cope with various types of masticatory forces [9,11].

Cementum is the heterogenic mineralized layer, which is deposited on the tooth dentin surface with a 50–300 μm thickness [12,13,14], and the biochemical compositions of cementum are similar to bone tissues with approximately 50% mineral and 50% organic components [15,16]. From the aspect of tissue functions, cementum plays the crucial role of anchoring PDL fibers onto tooth-root surfaces with Sharpey’s fibers, which are critical for periodontal functioning restorations [12,14]. Cementum is generally classified into two different types of acellular and cellular cementum tissues, according to the location and function of human teeth [13,14]. Briefly, the acellular cementum contributes to the tooth attachment of principal fibers of PDLs for periodontal functions, while cellular cementum is deposited around the apical regions of tooth roots for the positional adaptations of teeth to occlusion for periodontal repair [1,14,17]. 

Due to the tissue’s significance, such as the morphological and functional characteristics of cementum, various studies have been undertaken to understand and to attempt to develop cementum regeneration approaches for periodontal tissue integrations and functional PDLs [11,13,18]. Although cementogenesis is anticipated to lead to the promotion of a new fibrous tissue attachment process for periodontal tissue engineering, it still remains a major challenge to regulate the optimal dimension formations without ankylosis, the bone fusion to the tooth-root surfaces, and to develop the highly-predictable techniques for cementogenic procedure for PDL attachments [1,12,14]. 

### 1.2. Periodontal Disease and Treatments

Periodontal disease or periodontitis, which is a common inflammatory chronic disease, causes irreversible periodontal complex destruction, disconnection of principal fibrous ligamentous tissues, and consequent loss of barrier function [19,20,21]. The invasion of oral bacteria or the formation of dental biofilm (or the maturation of dental plaque) on tooth surfaces are mainly associated with pathogenesis of periodontal disease caused by host inflammatory responses [21,22]. In addition to periodontal pathogens, environmental and host genetic factors could also disturb severe periodontal manifestations to induce periodontitis [23]. In general, non-surgical periodontal treatments have been widely performed, such as scaling or root planing to mechanically remove periodontal pathogens, or anti-infective treatments to biochemically eliminate bacteria or microbial biofilm [24]. In the case of surgical treatments, the guided tissue regeneration (GTR)/guided bone regeneration (GBR) techniques are commonly employed for periodontal complex regenerations with osteoconductive/osteoinductive materials or bioactive biologics [5,25,26,27,28]. However, various therapeutic strategies for periodontal tissue regenerations are still too unpredictable and uncontrollable to form multiple tissue formation for fiber-mineral tissue complexes, spatial tissue compartmentalization with micron-scaled dimensions, specific angulation structures of PDLs, and tissue integrations for functioning restorations as tooth-supportive structures [5,24,29]. Therefore, severe cases of periodontitis around natural tooth structures could result in loosening teeth and would still be required after natural tooth extractions for dental prosthetics. 

Recently, regenerative strategies have been investigated for pre-clinical and clinical situations and developed for periodontal tissues, like implantable scaffolds or biologic delivery systems [30,31,32]. Major approaches in periodontal tissue engineering have focused on the development of bone substitutes or alveolar bone regeneration materials for dental prosthetic stabilities or natural tooth preservations [33,34]. However, the regeneration and configuration of PDL-cementum complexes still depends on biomechanical optimization during mastication or existing stem cell-like cell activations, like proliferation or differentiation in PDL-cementum interfaces for periodontal revitalization, following histophysiological adaptations of the periosteum [35,36]. In particular, due to the difficulties in 1) reconstituting 3D structures of PDLs and cementum along the tooth surfaces, 2) controlling specific orientations of fibrous connective tissues, and 3) guiding hierarchical multiple tissue formations within approximately 500 μm interfaces, it has remained challenging [18,24,37]. The described limitations cause unpredictable and unsatisfactory regenerations of tooth-supportive complex formations by the attachments of both mineralized and ligamentous tissues, as well as functional restorations of neogenic periodontal tissues [24,38]. In this review, recent advances are introduced with the individual 3D platforms for angularly engineered PDLs and the specific biomaterial fabrication for cementum regeneration with Sharpey’s fiber insertions.

## 2. PDL Regenerations with Angular Organization Using Topographic Approaches of 3D Platforms

Although multilayered scaffolds [32,39,40] and PDL cell sheet technology [2,41,42] have facilitated spatially compartmentalized hierarchical tooth-supportive structures with specific dimensions for individual periodontal tissues, it is challenging for fibrous tissues to be directionally controlled using micron-scaled, engineered architecture. In particular, topological strategies on 2D substrates have been actively developed to control the angular organizations of PDL cells [43,44,45] as well as other fibrous connective tissue cells [46,47]. Takahashi et al. fabricated a thermoresponsive brush surface using poly(*N*-isopropylacrylamide) (PIPAAm) to obtain selective patterns and regulate human dermal fibroblast alignments [47]. The results demonstrated that cultured fibroblasts on chemically patterned surfaces showed increased cell populations while maintaining the orientation for five days in vitro [47]. Moreover, engineered cell sheets could be detached from the fabricated culture dishes with the angular orientations and easily transplanted to target tissues or organs, which require the specific angulations of tissues [47].

**Figure 2:**
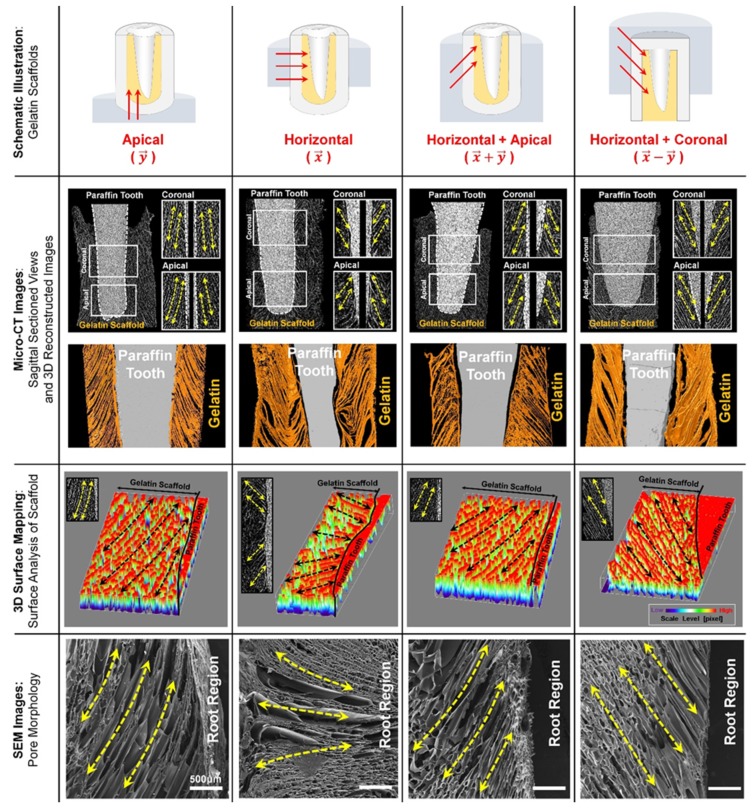
Freeze-casting method for longitudinal pore structures with controlled angulations. Depending on freezing directions (the first row), various angular organizations of internal pore architectures were created and analyzed using micro-computed tomographic images with both digitized 2D cross-sectional and 3D reconstructed images (the second row), 3D surface mapping to show angulations to the surface of the paraffin tooth (the third row), and scanning electron microscopic (SEM) images for morphologies (the fourth row). Adapted with permission from the reference [3].

Although there have been many efforts to control and regulate the orientations of fibrous connective tissue cells based on the developments of substrate topologies, they are significantly limited to investigate 3D platforms for the perpendicular/oblique orientations of PDLs to tooth-root surfaces. Recently, several techniques have been developed to physically control the orientations or angulations of PDLs using the fiber-guidable 3D microstructures of scaffolds, which could be created by directional freeze-casting, additive manufacturing, or soft lithographic methods [3]. The freeze-casting method with temperature gradients could simply create longitudinal pore architectures by ice crystal formation and control the directional internal architectures in gelatin hydrogels with predictability [3]. In particular, ice crystal growth kinetics by different freezing mold surfaces spatially created structural similarities to natural PDLs, 3–10 μm diameter of cementum-associated and 10–20 μm diameter of bone-associated PDL bundles [3]. After removing ice crystals from frozen gelatin scaffolds by freeze-drying, directionally longitudinal pore structures could have oblique orientations or radial angulations to the tooth-root surfaces (Figure 2). The study demonstrated that the freeze-casting method could create oblique PDL structures, which constitute approximately 70% of the PDL architectures, even though it is difficult to make other PDL structures with radial and horizontal orientations. In the results, the freeze-casted hydrogels can be the 3D platform in order to guide angularly organized PDLs with anatomically structural similarities to natural oblique PDL bundles [3].

**Figure 3:**
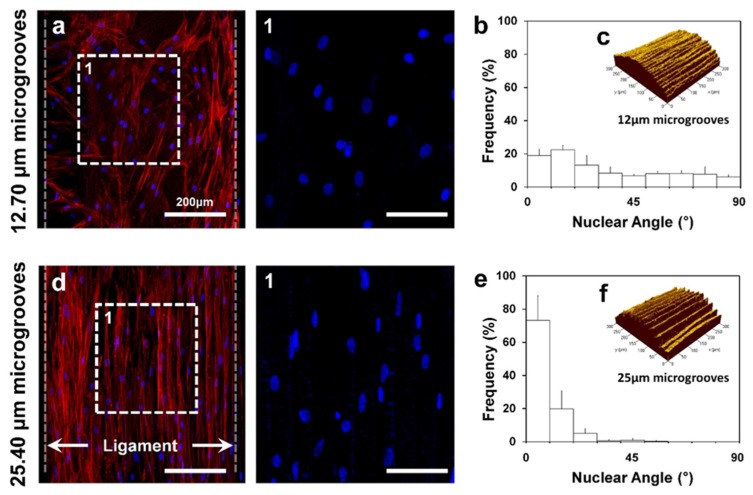
Cell orientation controls using different micro-groove intervals with 12.70 μm and 25.40 μm distance between grooves. After culturing human PDL cells for seven days, (**a**–**c**) 12.70 μm micro-groove intervals randomly organized cells but (**d**–**f**) 25.40 μm interval substrate guided highly aligned cells. Adapted with permission from the reference [8].

The additive manufacturing or 3D printing technique has been recently addressed to create micro-groove patterns on PDL scaffold surfaces with specific pattern intervals [8,32,37]. In general, the additive manufacturing technique can generate groove patterns on the features. Patterns are considered as topographical artifacts (or so-called stair-stepping errors), which should be removed to obtain smooth surfaces. Park et al. reinterpreted the artifactual patterns as micro-groove patterns with high predictability and developed three different angular patterns with optimal micro-groove intervals for human PDL cell alignments in manufacturing procedures. Additive layer thickness, which is reflected in micro-groove intervals, can be configured for the surface roughness and modeling speeds. To optimize intervals to regulate cell alignments, the study designed two types of micro-groove intervals (12.70 μm and 25.40 μm intervals between groove patterns) with in vitro experiments. The results showed that intervals were significantly affected for angular cell alignments and cell nucleus deformation, regardless of micro-groove patterns on the surfaces of scaffolds in seven-day cultures (Figure 3). That is, 12.70 μm micro-groove intervals showed a random organization of cells, while 25.40 μm intervals significantly aligned cells with parallel angulations to the reference direction for seven days (Figure 3) [8].

**Figure 4:**
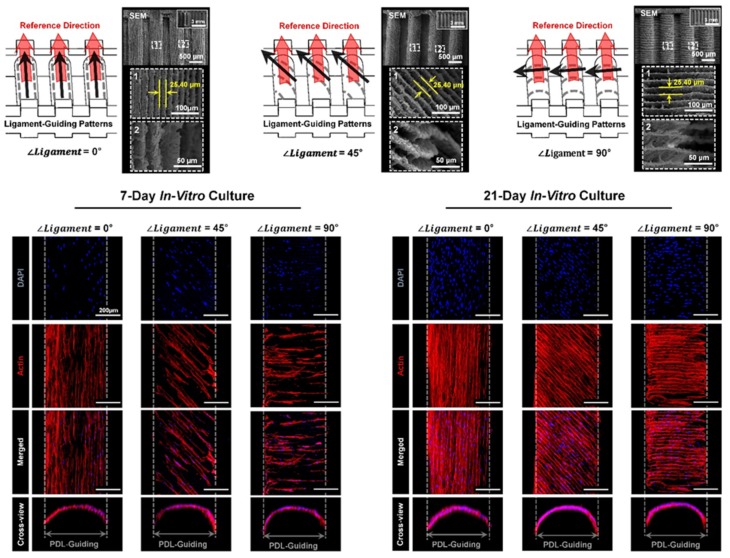
The scanning electron microscopic (SEM) images of micro-groove patterns on the surface of PDL architectures and fluorescence images of cell alignments in 7- and 21-day in vitro cultures for qualitative assessments. Three different micro-groove patterns facilitated angular organization of human PDL cells in 7- and 21-day cultivations. In particular, the micro-grooved topographies with 25.40 μm interval showed higher populations of cells as well as maintaining specific cell orientations. Adapted with permission from the reference [8].

Based on the interesting results of micro-groove intervals, PDL-guiding scaffolds were manufactured with three different angulated micro-groove patterns at 0°, 45°, and 90°, which could be parallel, oblique, and perpendicular to the reference directions, respectively (Figure 4) [8]. After the seeding and in vitro culturing of human PDL cells within scaffolds, different topographical-guiding microarchitectures on the surfaces of scaffolds could guide the orientations of cells with angles (0°, 45°, and 90°) in seven days and cell angulations cultured for 21 days were maintained with higher cell populations (Figure 4). Therefore, the layer thickness configured at the digital slicing and the manufacturing steps could be the critical factor to determine ligamentous cell orientations, which angulated micro-groove patterns could regulate using the 3D printing system. 

**Figure 5:**
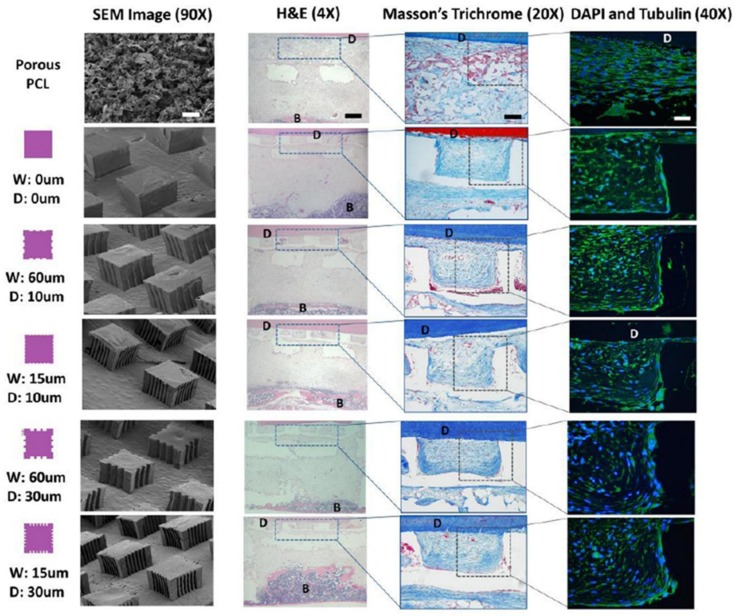
The surface characterizations of designed and manufactured micro-patterned poly-ε-caprolactone (PCL) films using scanning electron microscopy (SEM) and histological analyses of fibrous tissue formation following different patterns. The morphological significance of designed architectures was qualitatively analyzed using SEM following the design legends (first and second columns). For the histologies of PDL-guiding PCL films, hematoxylin and eosin (H&E) staining (third column), Masson’s trichrome staining (fourth column), and fluorescence staining using 4′,6-diamidino-2-phenylindole (DAPI; blue) and tubulin (green) staining (fifth column) showed fibrous tissue formation with collagen and cell alignments at six weeks. (D: dentin; B: bone). Adapted with permission from the reference [48].

In addition to 3D manufacturing techniques and architectures for PDL orientations, PDL-guidable micro-patterned 2D PCL films were fabricated by the soft lithographic technique, which created submicron-scaled topographies on PCL pillars for angular PDL organizations [48,49]. This study investigated the optimization of the width and depths of micro-groove patterns to control the orientations and thickness of regenerated PDL fibrous bundles in vitro and in vivo [48]. The results demonstrated that alignments of human PDL cells with newly-formed collagenous bundle orientations corresponded with topographical characteristics by the lithographic design and manufacture, but large inter-pillar spaces like 400 μm did not allow the formation of oriented fibrous tissue or develop the density of collagen bundles (such as fiber-structure thickness) [48]. Therefore, the study demonstrated that the optimal dimensions and geometries of PDL-guiding scaffolds were critically required for angular PDL and collagenous fiber bundle formations (Figure 5) [48]. As the results of the subcutaneous transplantation model showed, the designed dimensions of the width and the depth of the micro-grooves significantly affected cell alignment and collagen bundle organization between the bone scaffold and the dentin segment surface (Figure 5) [48]. In addition, the chemical vapor deposition (CVD) technique was applied to fabricate the surfaces of polymer-based PDL-guiding films with growth factors, which could promote tissue regeneration within compartments for different periodontal tissues [49]. The growth factor-associated periodontal scaffolds could synergistically enhance periodontal tissue regenerations like bone, PDL, and limited cementum around exposed tooth-root surfaces in the in vivo periodontal fenestration defects of the rat models [49].

**Figure 6:**
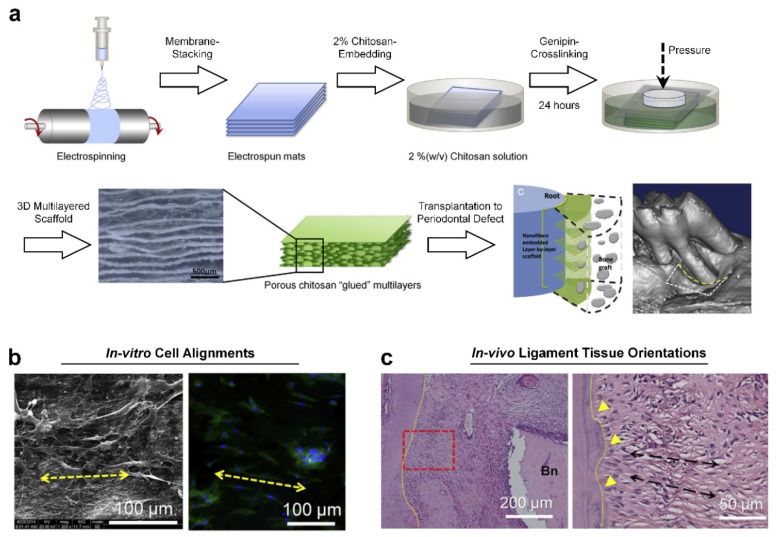
The electrospun nano-/micro-fibrous 3D scaffolds for engineered periodontal ligament (PDL) orientations. (**a**) The schematic illustration shows 3D stacked scaffold creation using the electrospinning fabrication, and chitosan solution was facilitated for 3D structures with the genipin crosslinking agent. For in vivo transplantation, the periodontal defects around the mesial root of the first molar tooth were surgically created in a rat model. (**b**) In vitro, the seeded cells showed polarized cytoskeletons and morphological alignments using scanning electron microscope (SEM) and fluorescence microscope images (Blue: DAPI and Green: actin). The arrowed yellow-dash line indicates directions of aligned cells. (**c**) By the hematoxylin and eosin (H&E) staining method, PDL-like fibrous connective tissues were formed with the perpendicular orientations to the tooth-root surface in two months. The yellow triangles indicate the limited but newly formed cementum-like tissue layer and the arrowed back-dash lines indicate the orientations of fibrous tissues. (Bn: Bone). Adapted with permission from the reference [51].

In addition to the soft lithographic strategy, the electrospinning fabrication technique has been widely utilized to manufacture random or highly aligned nano-/micro-fibrous membranes in dental tissue engineering applications such as periodontal regeneration [50,51,52] or pulp regeneration [53,54,55]. In particular, the electrospun 2D membranes with unidirectional alignments or controllable-aligned patterns of nano-fibers were investigated for specific cell orientations and migrations to regulate cellular responses to designed microenvironments [56,57]. However, there is the limitation to create 3D directional internal architectures to regulate cell or tissue orientations with structural similarities to PDLs because periodontal connective tissues have the spatially perpendicular or oblique angulations to tooth-root surfaces as described above [51,58]. Jiang et al. recently developed 3D architectures, which were stacked using electrospun nano-fibrous membranes and assembled with crosslinked chitosan hydrogels (Figure 6a) [51]. After the blended biopolymeric material (poly-ε-carpolactone and polyethylene glycol; PCE) was electrospun for nano-fibrous membranes, the PCE membranes were stacked layer-by-layer and assembled in chitosan solution with the genipin crosslinking agent (Figure 6a). In the results, the cells seeded into scaffolds were directionally aligned with polarized morphologies in vitro (Figure 6b). PDL-like fibrous connective tissues were angularly reorganized perpendicularly or obliquely, as well as cell infiltration, into the 3D scaffolds in in vivo (Figure 6c) [51]. 

## 3. Cementum Regeneration on the Tooth-Root Surface Using Biomaterial-Based 3D Engineered Environments

Cementogenesis, or cementum regeneration, is a critical procedure for the new attachment process and structural integration of multiple periodontal tissues to generate tooth-supportive functioning restoration like the anchorage of collagenous fibrous tissues, PDLs [5,12,14,37]. Various biomaterials have been investigated to induce the interfacial tissue formation between the tooth dentin and PDL fibrous tissues using 3D biopolymeric scaffolds [59]. The poly(lactic-co-glycolic acid) (PLGA) scaffolds played a key role in carrying cementoblastic cells and cementogenesis-promoting biologics, like platelet-derived growth factor-BB (PDGF-BB), for cementoblastic cell activation and cementogenesis in in vivo environments [60,61]. However, most investigations in cementogenesis have mainly focused on the developments of biopolymeric carriers for delivering bioactive molecules [60,62,63,64,65,66], as well as physiological/pathological adaptations and cementogenic differentiations of dental stem cells in micro-niches [20,67,68,69], rather than geometric or architectural regulations for the cementogenesis. It could be difficult for the cementum regeneration to spatiotemporally control mineralized layer depositions on the tooth-root surfaces in micron-scaled thickness, so it still depends on the biological activations of transplanted stem cells or biologic-activated host cells/tissues. 

**Figure 7:**
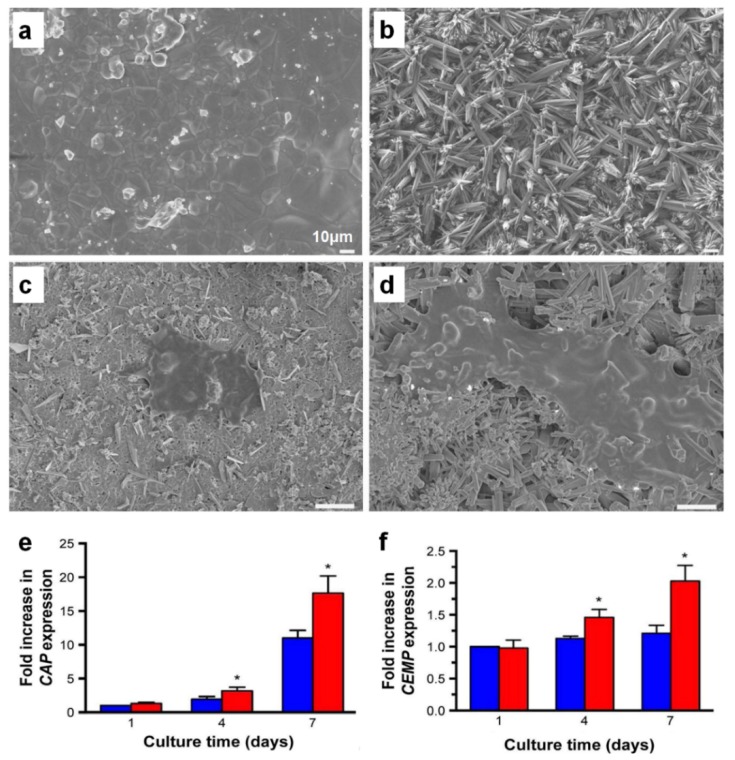
The morphological characterizations of the traditional hydroxyapatite and the nano-/microstructured hydroxyapatite with cementogenic expression levels in vitro. (**a**,**c**) The traditional hydroxyapatite bioceramic material surfaces were characterized by a scanning electron microscope (SEM) before and after the human PDL stem cell seeding. (**b**,**d**) The nano-/microstructure characterized hydroxyapatite materials were evaluated using SEM before and after human PDL stem cell seeding. (**e**,**f**) The cementogenic expression levels were quantitatively assessed with cementum attachment protein (CAP) and cementum protein (CEMP). The fabricated hydroxyapatite surfaces (red columns) showed statistically significant differences from the traditional (blue columns). (* *p* < 0.05). Adapted with permission from the reference [70].

Recently, the different topologies or the biodegradability of biomaterials have been investigated to activate the canonical Wnt signaling or Wnt/β-catenin signaling pathway, which is involved in inducing cementogenesis and osteogenesis in the periodontal tissue development or regeneration. Mao et al. demonstrated that the bioceramic materials fabricated to create nano-/microstructures could highly promote cementogenic differentiation using human PDL cells [70], which could differentiate cementoblast-like cells and form a hierarchically compartmentalized structure like cementum-PDL constructs [71,72,73]. While traditional calcium phosphate (CaP)-based bioceramic materials have good osteoconductivity to induce bone formation, the lack of osteoinductivity is required to improve cementum formation for which stem cell-like cells or various biologics are commonly contributed [74,75]. Therefore, nano-/microstructures with 3D topographic specificity were recently developed to promote the cementogenic differentiation of human PDL stem cells as well as cell attachment on the fabricated surface (Figure 7) [70]. In particular, the canonical Wnt signaling pathway, which is involved in the physiological process of mineralized tissue development [76], was activated on the nano-/microstructured surfaces in order to enhance the cementogenic differentiation of human PDL stem cells [70]. 

**Figure 8:**
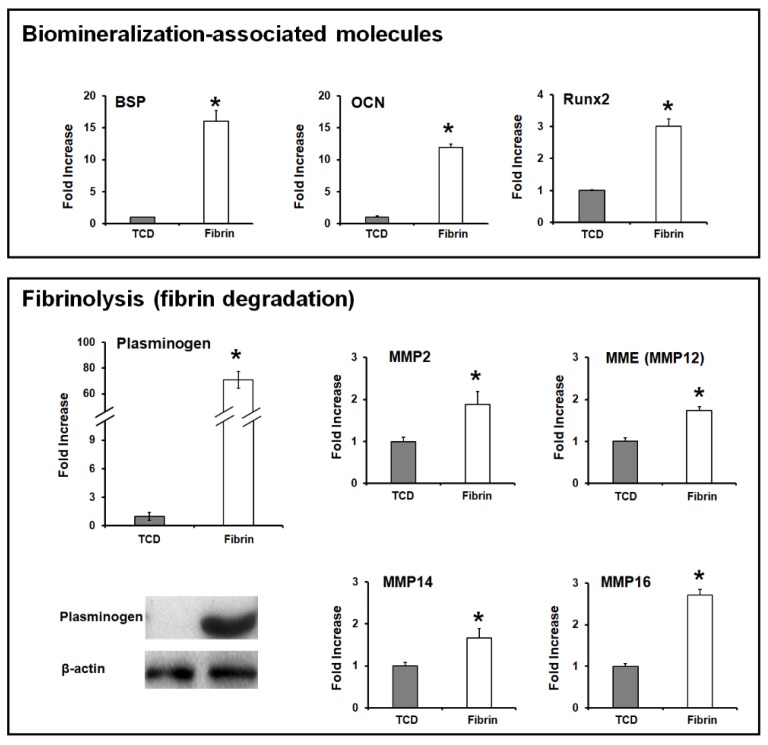
Cementoblast cell activations for biomineralization and fibrinolysis. The fibrin hydrogel matrices for the cementoblast cell culture could activate the canonical Wnt signaling pathway to promote the higher expression of biomineralization-associated molecules like bone sialoprotein (BSP), osteocalcin (OCN), and Runx-related gene 2 (Runx2) (upper panel). Simultaneously, cementoblast cells could have a significantly high expression level of plasminogen on fibrin hydrogel matrices rather than tissue culture dish (TCD) environments. Moreover, expressions of fibrinolytic elements like matrix metalloproteinases (MMPs) increased during cementoblast cell cultivation on fibrin hydrogel matrices (lower panel). * *p* < 0.05. Adapted with permission from the reference [82].

Although the canonical Wnt signaling pathway has been known to significantly contribute to the tooth morphogenesis at the tooth development stage and the activation of the cementogenic differentiation of PDL cells [77,78,79,80,81], cementoblast cells in fibrin hydrogel matrices simultaneously increase plasminogen expression, which promotes matrix degradation and cell apoptosis in fibrin hydrogel (Figure 8) [79]. Rahman et al. investigated interactive fibrinolysis with the biomineralization process during OCCM30 cell (mouse cementoblast cells) cultivations [79]. Compared with conventional tissue culture dishes (TCDs), the OCCM30 cells in the fibrin hydrogels had high expression levels of biomineralization-associated molecules like bone sialoprotein (BSP), osteocalcin (OCN), and Runx-related gene 2 (Runx2) with statistical significances (* *p* < 0.05; Figure 8). Based on the results, fibrin hydrogel matrices could critically enhance cementogenic or osteogenic differentiation, but the higher expression level of the plasminogen activator by cementoblasts than by the fibrin matrices simultaneously led to fibrinolysis (fibrin degradation), loss of cementoblast-fibrin matrix interactions, and subsequent cell apoptosis in Figure 8 [12,82]. 

**Figure 9:**
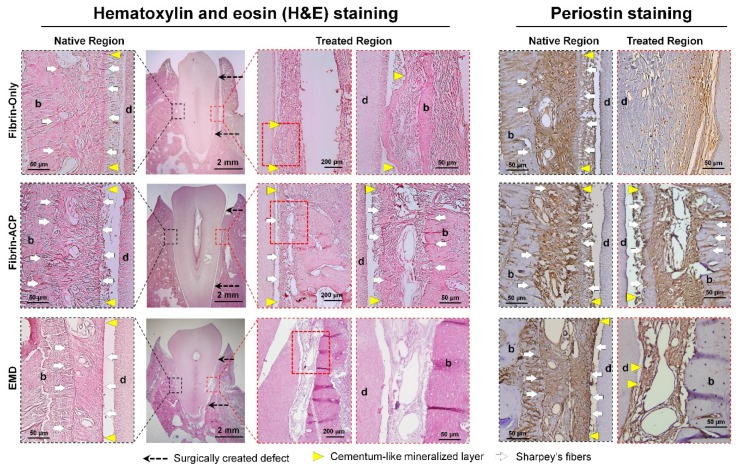
Histological and immunohistological analyses of cementum formation and Sharpey’s fiber insertions to bone and newly formed cementum tissues. Compared with fibrin-only (unmodified fibrin hydrogel) and enamel matrix derivative (EMD) groups, the fibrin-ACP (modified fibrin hydrogel) facilitated the regeneration of periodontal tissues such as cementum on tooth-root surfaces, PDL, and the alveolar bone. More critically, the fibrin-ACP promoted Sharpey’s fiber formations and insertions into the cementum layers and alveolar bone surfaces, indicated by white arrows. (d: tooth-root dentin; b: bone). Adapted with permission from the reference [12].

Moreover, when the fibrin hydrogel was used as the substrate to culture cementoblasts and osteoblasts in vitro, the apoptosis of cementoblasts was quantitatively and qualitatively assessed with a statistically significant difference from osteoblasts [12]. The higher expression level of the plasminogen activator by cementoblasts rather than osteoblasts to the fibrin matrices led to fibrinolysis (fibrin degradation), loss of cementoblast-fibrin interactions, and subsequent cell apoptosis [12,82]. Therefore, ε-aminocaproic acid (ACA), which is an inhibitor of the plasminogen activator, was utilized to inhibit fibrinolysis and maintain fibrin fibril structures for cementoblast differentiation, mineralization, and tissue maturation for periodontal tissue regeneration and integrations [12]. Based on the in vitro findings that modified fibrin hydrogel with ACA molecules, facilitated cementoblast cell viability, and promoted differentiation for mineralization, the in vivo periodontal regeneration study was designed with fibrin, modified fibrin, and enamel matrix derivative (EMD). After the creation of the Class II furcation defect model in beagle dogs, three different groups were placed to promote cementum, PDL, and alveolar bone formations with quantitative and qualitative analyses [12]. Interestingly, the modified fibrin matrices could induce bone regeneration and mineral deposition on the tooth-root surface, even though only limited PDLs and orientations of fibrous connective tissues were observed between cementum and bone surfaces (Figure 9) [12].

Interfaces of cementum-PDL and bone-PDL in the modified fibrin hydrogels showed Sharpey’s fiber insertions, which could have fiber anchorages between different mineralized tissue surfaces and re-functionalizing potential as tooth-supportive complexes [12]. It is still hard to demonstrate that the modified fibrin hydrogel could allow the functional restorations of periodontal tissues because the structural hierarchies have strongly required mineralized tissue formations with specific orientations of PDLs. However, the fibrin hydrogel modification and fabrication could be investigated for regenerative effects for compartmentalized tissue formations with the induction of Sharpey’s fiber formations and insertions.

**Figure 10:**
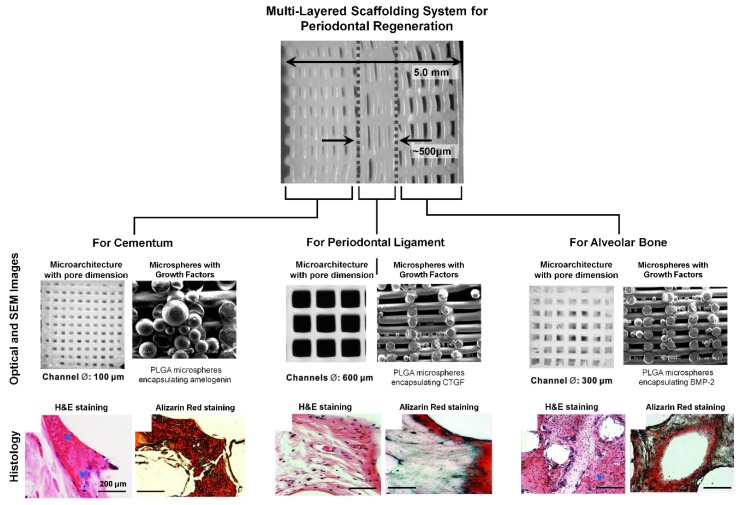
Optical and scanning electron microscopic (SEM) images of 3D printed scaffold and histological analyses. Specific pore dimensions with the spatial compartmentalization were designed for multiple tissue formation and infiltration, such as cementum, periodontal ligament (PDL), and the alveolar bone. In particular, poly(lactic-glycolic) acid (PLGA) microspheres were tethered with different bioactive molecules to promote individual tissue growth like the amelogenin for cementum, the connective tissue growth factor (CTGF) for PDL, and bone morphogenetic protein-2 (BMP2) for the alveolar bone. The histology analyses with hematoxylin and eosin (H&E) staining and Alizarin red staining showed the mineralized tissue formation and fibrous tissue insertion into the mineralized tissues in vivo, which was performed with the subcutaneous immunodeficient mouse model [59].

Although various scaffolding approaches have been investigated to regulate cementogenesis and mineralized layer formation with the integration of fibrous connective tissues, the spatial provision for cementum formation within micron-scaled compartmentalized interfaces is still challenging. Lee et al. used the 3D printing system to manufacture multi-layered structures for the periodontal complex as well as specific internal pore architectures with different dimensions for individual tissues [59]. In addition, three different recombinant human growth factors (amelogenin for cementogenesis, connective tissue growth factor (CTGF) for PDL regeneration, and bone morphogenetic protein-2 (BMP2) for osteogenesis) were encapsulated using PLGA microspheres spatially tethered to each region (Figure 10) [59]. In this study, different pore-microstructures and types of bioactive molecules in a single tissue engineering scaffold strongly correlated with multiple tissue regenerations [59]. More interestingly, aligned fibrous tissue structures limitedly showed the fibrous insertion and anchorage to mineralized layers in the PDL region of the scaffold, so the internal pore-structures could be characterized for periodontal tissue integrations and compartmentalized architectures with bioactive molecules could lead to the formation of fibrous-mineralized tissue constructs (Figure 10) [59].

## 4. Future Prospects for Biomaterial-Based Periodontal Tissue Engineering

3D printing techniques have been employed to develop various scaffolding designs for periodontal tissue regeneration for pre-clinical and clinical scenarios to spatiotemporally compartmentalize individual tissues such as cementum, PDL, and the alveolar bone [30,31,32]. However, the technology is still challenged to create micron-scaled tissue compartments for the individual periodontium (especially, cementum and PDLs) and to control spatiotemporal tissue formations with their unique individual characteristics [5,37]. This review highlights the recently developed 3D platforms to guide orientations of engineered PDLs and promote cementogenesis as the interfacial tissue layer to induce tissue integrities by biomaterial modifications for the revitalization of periodontal complexes in vitro and in vivo. Although these novel techniques need to integrate existing techniques like 3D printing, cell sheet engineering, or cell spheroid approaches, they will be the principal and predominant strategies for the new paradigm of periodontal regenerative medicine with greater predictability and high controllability.

## Abbreviations

PDL	Periodontal ligament
CEJ	Cementoenamel junction
GTR	Guided tissue regeneration
3D	3-dimensional
Micro-CT	Micro-computed tomography
SEM	Scanning electron microscopy
PCL	Poly-ε-caprolactone
H&E	Hematoxylin and eosin
PCE	Poly-ε-caprolactone and polyethylene glycol
CVD	Chemical vapor deposition
TCD	Tissue culture dish
BSP	Bone sialoprotein
OCN	Osteocalcin
Runx2	Runx-related gene 2
MMPs	Matrix metalloproteinases
EMD	Enamel matrix derivative
ACA	ε-aminocaproic acid
ACP	ε-aminocaproic acid incorporated with chitosan nanoparticle
CaP	Calcium phosphate
CAP	Cementum attachment protein
CEMP	Cementum protein
PLGA	Poly(lactic-co-glycolic acid)
CTGF	Connective tissue growth factor
BMP-2	Bone morphogenetic protein-2
PDGF-BB	Platelet-derived growth factor-BB
